# Calycosin attenuates the inflammatory damage of microglia induced by oxygen and glucose deprivation through the HMGB1/TLR4/NF-κB signaling pathway: Calycosin attenuates OGD/R damage in microglia

**DOI:** 10.3724/abbs.2023125

**Published:** 2023-08-01

**Authors:** Xiang Li, Xin Yang, Huiling Lu, Wenbo Wang, Le Cai, Jian Chen, Yong Wang

**Affiliations:** 1 Key Laboratory of Tumor Immunology and Microenvironmental Regulation Guilin Medical University Guilin 541199 China; 2 Department of Pathology and Physiopathology Guilin Medical University Guilin 541199 China; 3 Department of Neurosurgery Nanxishan Hospital of Guangxi Zhuang Autonomous Region Guilin 541002 China; 4 Department of Physiology Guilin Medical University Guilin 541199 China

**Keywords:** calycosin, HMGB1/TLR4/NF-κB signaling pathway, microglia, oxygen and glucose deprivation, neuroinflammation

## Abstract

Stroke seriously threatens human life and health worldwide, but only very few effective stroke medicines are currently available. Our previous studies have indicated that the phytoestrogen calycosin exerts neuroprotective effects in cerebral ischemia and reperfusion injury rats. Therefore, the objective of this study is to further explore the protective effect of calycosin on inflammatory injury in microglia after oxygen-glucose deprivation/reoxygenation (OGD/R) and to clarify whether its protective effect is related to the HMGB1/TLR4/NF-κB signaling pathway. Here, the OGD/R model of rodent microglia is established *in vitro* to simulate cerebral ischemia-reperfusion injury. Through the CCK-8 test, ELISA, qRT-PCR, and western blot analysis, we find that the activity of microglia is decreased, the expressions of HMGB1 and TLR4 and the phosphorylation of NF-κB (p-NF-κB) are increased, and the releases of the inflammatory factors IL-6, IL-1β, and TNF-α are increased after OGD/R. Pretreatment with calycosin could ameliorate these states, increase cell viability, reduce HMGB1, TLR4 and p-NF-κB expression, and reduce inflammatory cytokine production. In addition, the effect of calycosin is similar to that of TAK-242 (an inhibitor of TLR4), and the effect of the combined treatment is better than that of the single treatment. The results indicate that calycosin protects microglia from OGD/R injury and reduces the inflammatory response. Calycosin might alleviate cerebral ischemia-reperfusion injury by inhibiting the HMGB1/TLR4/NF-κB pathway.

## Introduction

The high mortality and disability rates of stroke cause a serious burden to families and society and have made stroke one of the most important diseases threatening human life. The incidence of ischemic stroke accounts for 80% of the incidence of stroke. When ischemic stroke occurs, the primary treatment method is to restore blood flow in time. However, after a period of ischemia and hypoxia in the brain tissue, reperfusion not only fails to restore its function but also further exacerbates the damage, which is known as cerebral ischemia-reperfusion injury (CIRI).

Activation of the inflammation-related signaling pathway is the basis of transformation from ischemic injury to inflammatory injury after cerebral ischemia-reperfusion. The high mobility group box 1 (HMGB1)/toll-like receptor 4 (TLR4)/nuclear factor-kappa B (NF-κB) signaling pathway is involved in multiple cellular processes, such as inflammation, apoptosis, autophagy, and cell survival [ 1– 3].However, the mechanism and regulation of this pathway in CIRI are currently not well understood.

Microglia, as innate immune cells of the central nervous system, mediate the neuroinflammatory response of CIRI [4]. Acute cerebral ischemia can lead to cytotoxic edema in neural and glial cells and generate reactive oxygen species (ROS), cytokines, and other inflammatory mediators, such as tumor necrosis factor-α (TNF-α), interleukin-1β (IL-1β), IL-6, matrix metalloproteinases (MMPs), and nitric oxide synthase (iNOS). When microglial cells switch from a resting state to a proinflammatory state, they can release many inflammatory mediators, increase vascular permeability, and promote neuronal cell death [5]. Therefore, inhibiting the release of proinflammatory factors from microglia may be an important way to alleviate cerebral ischemia-reperfusion injury.

As an isoflavone phytoestrogen, calycosin is one of the main active components isolated from the traditional Chinese medicine *Radix Astragali* [6]. Numerous studies have proven that calycosin has antitumour, antioxidation, anti-inflammatory, and neuroprotective properties [ 7 – 10]. Our previous research also found that calycosin exerts neuroprotective effects against ischemic brain injury in middle cerebral artery occlusion (MCAO) rats by inhibiting neuronal apoptosis, autophagy, and inflammatory processes [11]. Nevertheless, whether the underlying anti-inflammatory mechanism of calycosin in the protection of CIRI is relevant to the HMGB1/TLR4/NF-κB signaling pathway requires further investigation.

In this study, we aimed to explore the roles of the HMGB1/TLR4/NF-κB signaling pathway in OGD/R microglia. Based on this inflammatory signaling pathway, we hoped to further elucidate the underlying mechanisms of the neuroprotective effect of calycosin in CIRI.

## Materials and Methods

### Chemicals and reagents

Calycosin (C _16_H _12_O _5_, purity>98.0%; Yuanye Biotechnology, Shanghai, China) was dissolved in dimethyl sulfoxide (DMSO; Sigma Aldrich, St Louis, USA) and stored at 4°C prior to use. Enzyme-linked immunosorbent assay (ELISA) kits were obtained from Fankew (Shanghai, China). The specific primers were synthesized by Gene Create (Wuhan, China). TAK-242 was purchased from MedChemExpress (Monmouth Junction, USA), dissolved in DMSO and stored at ‒20°C or ‒80°C.

### Cell culture

Primary rat microglial cells were isolated and cultured according to a previously described method [12], with minor modifications. In brief, primary microglia were derived from mixed glia in the cortex of neonatal Sprague-Dawley (SD) rats (1‒3 days old) and isolated using the “shaking off” method. After dissection of the cerebral hemispheres, the meninges were removed. The cortical tissues were minced in ice-cold dissection solution and then dissociated in a sterile petri dish containing DMEM/F12 (Boster, Wuhan, China) supplemented with trypsin (Solarbio, Beijing, China) and DNase I (Sigma Aldrich). Digestion was terminated using DMEM/F12 media containing 10% fetal bovine serum (FBS; Lonsera, Shanghai, China). After centrifugation at 200 *g* for 5 min, the supernatant was discarded, and the cells were plated at 1.5×10 ^6^ cells/mL in 75 cm ^2^ flasks coated with 0.1 mg/mL poly-L-lysine. The cultures were maintained in DMEM/F12 supplemented with 10% FBS and 1% penicillin/streptomycin (Solarbio). The cells were kept in a humidified atmosphere of 5% CO _2_ and 95% air at 37°C. The medium was refreshed every 2‒3 days. When the mixed glial cultures reached confluence (on the 9th–10th days *in vitro*), flasks were shaken for 2 h at 350 rpm at 37°C, and floating microglia were collected and seeded onto poly-L-lysine-coated petri dishes. Then, fresh medium was added for further culture, and the fluid was changed every 2‒3 days. The purity of the primary microglial cells was confirmed by immunofluorescence staining using its specific marker Iba1.

BV2 cells (Kunming Cell Bank of Chinese Academy of Sciences, Kunming, China) were cultured in high glucose phenol red-free RPMI-1640 medium (Giboco, New York, USA) containing 10% FBS and 1% penicillin-streptomycin), and HAPI cells (Otwo Biotech, Shenzhen, China) were cultured in high glucose phenol red-free DMEM (Boster) containing 10% FBS and 1% penicillin- streptomycin. Both cell lines were cultured at 37°C, 5% CO _2_, and saturated humidity. The cells were passaged for 2‒3 days, and the cells with good shape and at logarithmic growth phase were used for the subsequent experiments.

### Immunofluorescence staining

Primary microglial purity was assessed by immunofluorescence staining with the specific marker Iba1. To achieve this, primary microglial cells were cultured on coverslips for 2 days. Subsequently, the cells were washed three times with PBS for 3 min each time, fixed for 20 min at room temperature using 4% paraformaldehyde, and then subjected to permeabilization with 0.5% Triton X-100 in PBS for 20 min. Nonspecific binding was prevented by blocking the cells with 5% BSA for 0.5 h. Then, the microglial cells were incubated overnight at 4°C with rabbit anti-Iba1 monoclonal antibody (1:100; Proteintech, Wuhan, China) in blocking solution. After three times wash with PBS, the microglial cells were treated with FITC-conjugated goat anti-rabbit IgG (1:200; Beyotime Biotech, Shanghai, China) for 2 h at room temperature, and the nuclei were stained with DAPI (Beyotime Biotech). Fluorescence images were obtained using an inverted fluorescent microscope (Olympus Corporation, Tokyo, Japan). The quantity of Iba1 ^+^ and DAPI ^+^ cells was counted to evaluate microglial purity.

### Oxygen-glucose deprivation/ reoxygenation (OGD/R) modelling

The microglia OGD/R model was established as previously described with minor modifications [13]. Briefly, well-grown BV2, HAPI, and primary microglial cells were pretreated with calycosin for 24 h, and then, the medium was replaced by sugar-free Earle’s balanced salt solution. Then, the cells were placed in a portable cell hypoxia/anoxic chamber (Billups-Rothenberg, Delmar, USA) with 95% N _2_ and 5% CO _2_ at 37°C for an appropriate length of time, which completed the oxygen-glucose deprivation model to simulate ischemia and hypoxia. The OGD time of BV2 and primary microglial cells was 4 h, and that of HAPI cells was 2 h. After these OGD periods, Earle’s balanced salt solution was replaced by the corresponding medium and cells were placed in a 37°C, 5% CO _2_ incubator, which completed the oxygen and glucose restoration (OGR) to simulate reperfusion. Subsequent experiments were performed after 24 h of OGR. In the control group, the cells underwent a similar procedure but did not undergo OGD/R.

### Cell viability assay

Calycosin was dissolved in DMSO to prepare a stock solution with a concentration of 0.1 M, stored at 4°C, and diluted with the cell culture medium as needed. Cell counting kit-8 (CCK8) assay was performed to measure cell viability. According to the cell growth rate, the corresponding number of well-grown BV2 and HAPI cells were seeded in 96-well plates. Different doses of calycosin were used for pretreatment for 24 h. After BV2 cells underwent OGD for 4 h or HAPI cells underwent OGD for 2 h, both of these types of cells were returned to normoxic and normoglycemic conditions for 24 h. Immediately following this, CCK8 solution (CK04; Dojindo, Kumamoto, Japan) was added to the wells to be tested, and the plates were placed in a 37°C, 5% CO _2_ cell incubator for 4 h. Then, the optical density (OD) values of each well were measured at 450 nm with a microplate reader (BioTek Instruments, Winooski, USA).

### Western blot analysis

The cells were lysed using RIPA lysis buffer (Solarbio) to extract the total proteins. According to the instructions of the BCA Protein Assay Kit (Beyotime Biotech, Shanghai, China), the protein concentration was detected and adjusted with RIPA lysis buffer and SDS-PAGE loading buffer (Solarbio) to maintain the same protein content in each group. Equal amounts of protein were separated by 10% SDS-PAGE; the samples were then transferred onto polyvinylidene difluoride (PVDF) membranes (Bio-Rad Laboratories, Hercules, USA). To block nonspecific binding, 5% skimmed milk was used for the incubation. Then, within the range given in the instructions, the appropriate concentration of the primary antibody was incubated on the PVDF membrane overnight at 4°C: anti-β-actin (TA-09; 1:2000; ZSGB-Bio, Beijing, China), anti-NF-κB p65 (1:1000; Cell Signaling Technology, Danvers, USA,), anti-phosphor-NF-κB p65 (1:1000; Cell Signaling Technology), anti-phosphor-IκBα (1:10,000; Abcam, Cambridge, UK), and anti-IκBα (1:10,000; Abcam). After three times wash with TBST for 10 min each, the membranes were incubated with horseradish peroxidase-conjugated secondary antibody for 1 h at room temperature. After the strips were washed again with TBST for three times, an enhanced chemiluminescence (ECL) reagent kit (Affinity Biosciences, Beijing, China) was added dropwise, and the result was visualized with the ChemiDoc ^TM^ XRS (Bio-Rad) system. Then, the grey values of the bands were analysed using Image Lab Software.

### Enzyme-linked immunosorbent assay (ELISA)

The levels of HMGB1, TNF-α, IL-1β, and IL-6 in the supernatants of BV2 and HAPI cells were determined using their corresponding ELISA kits (Fankew) strictly according to the manufacturer’s instructions. ELISA was also used to determine the TNF-α, IL-1β, and IL-6 levels in the supernatant of the primary microglia. Briefly, the supernatants of the cell culture medium as well as the standard samples were added into the wells of the microtiter plates. After incubation at 37°C, washing, color development and termination of the reaction, the OD values of the wells were measured at 450 nm with a microplate reader (BioTek Intruments). Finally, the levels were calculated using the standard curves.

### Quantitative real-time PCR (qRT-PCR)

Total RNA was extracted strictly following the instructions of the RNA simple total RNA kit (Tiangen Biotech, Beijing, China), and the concentration and purity of the obtained RNA were checked. The total RNA was then reverse-transcribed into cDNA using reverse transcription kit (MonScript™ RTIII All-in-One Mix with dsDNase, MR05101S; Monad Biotech, Suzhou, China) according to the manufacturer’s instructions. qRT-PCR was conducted on an ABI PRISM 7500 Sequence Detector System (Applied Biosystems, Foster City, USA). Total volume for reaction system was 20 μL, including 10 μL SYBR Green qPCR Mix, 0.4 μL of each forward and reverse primers, 8.2 μL of double distilled water, and 1 μL of template cDNA. The reaction conditions were as follows: pre-denaturation at 95°C for 30 s (1 cycle); denaturation at 95°C for 10 s, annealing at 55°C for 10 s, extension at 72°C for 30 s (40 cycles); one cycle of product melting by system default. The specific primers are listed in Table 1. The relative gene expression was qualified via the 2 ^‒∆∆CT^ approach, and *β-actin* was used as a normalization control.

**Table:** **Table 1** List of primers used for qRT-PCR

Cell	Gene	Forward sequence (5′→3′)	Reverse sequence (5′→3′)
BV2	*TLR4*	CAGAATGAGGACTGGGTGAGA	CTGTAGTGAAGGCAGAGGTGA
*HMGB1*	AGGCTGACAAGGCTCGTTATG	GATTTTGGGGCGGTACTCAG
	*β-actin*	TCATCACTATTGGCAACGAGC	AACAGTCCGCCTAGAAGCAC
HAPI	*TLR4*	ATGAGGACTGGGTGAGAAACG	GTTGGCAGCAATGGCTACAC
*HMGB1*	GGATGACAAGCAGCCCTATG	TCTTCTCAGCCTTGACCACC
	*β-actin*	TGTCACCAACTGGGACGATA	GGGGTGTTGAAGGTCTCAAA

### Molecular docking

To analyse the binding affinities and modes of interaction between the drug candidates (TAK-242, calycosin) and their target TLR4, AutodockVina 1.1.2, a protein-ligand docking software, was employed. The molecular structures of TAK-242 (Compound CID: 11703255) and calycosin (Compound CID: 5280448) were retrieved from the PubChem Compound database ( https://pubchem.ncbi.nlm.nih.gov/). The 3D coordinates of TLR4 (mouse: Q9QUK6, rat: Q9QX05) were downloaded from UniProt ( https://www.uniprot.org/). PyMOL and MOE software were used to visually analyse and assess the molecular interactions of TAK-242 and calycosin with TLR4. In addition, the root mean square deviation (RMSD) of the ligand molecule was used as a parameter to determine the accuracy of the docking, and the setting with an RMSD<4 Å is denoted as the threshold to conform to ligand molecules.

### Statistical analysis

All data are presented as the mean±standard deviation (SD). The statistical significance was evaluated using a two-tailed Student’s *t* -test or one-way analysis of variance (ANOVA) with a *post hoc* least significant difference (LSD) test. All of the statistical analyses were performed and diagrams were made using GraphPad Prism 8.0. The significance level was set at *P*<0.05.

## Results

### Effect of calycosin on microglial activity after OGD/R *in vitro*

**Figure 1:**
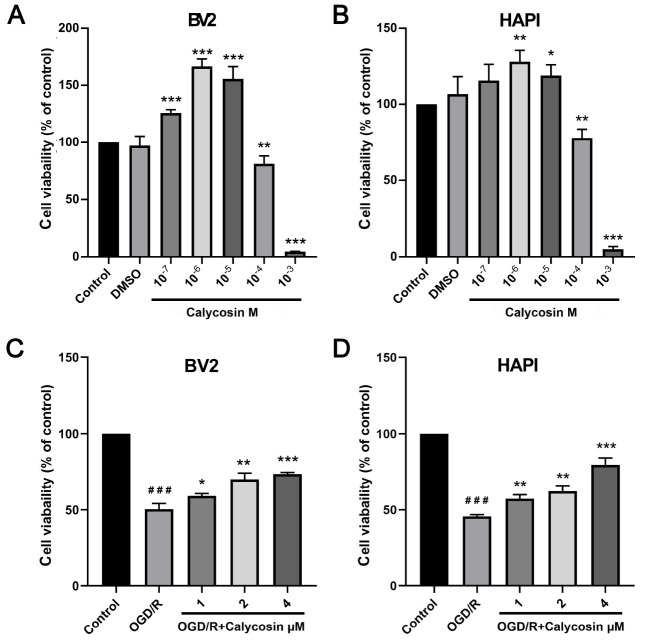
Figure 1 Effects of different concentrations of calycosin on microglial activity and microglial activity after OGD/R The cell activity was measured by CCK8 assay. (A) Effects of different concentrations of calycosin on BV2 cells. (B) Effects of different concentrations of calycosin on HAPI cells. (C) Effects of different concentrations of calycosin on BV2 cells after OGD/R. (D) Effects of different concentrations of calycosin on HAPI cells after OGD/R. Data are expressed as the mean±SD, n ≥ 3. *P<0.05, **P<0.01, ***P<0.001 vs the control group (A,B) or OGD/R group (C,D). ###P<0.001, the OGD/R group vs the control group (C,D).

To explore the effects of different concentrations of calycosin on microglia after OGD/R, CCK8 assay was used to measure the activity of microglia in this study. The results demonstrated that, compared with the control group, calycosin significantly increased BV2 and HAPI cell viability at concentrations between 10 ^‒7^ M and 10 ^‒5^ M, peaking at a concentration of approximately 10 ^‒6^ M ( Figure 1A). It was also observed that calycosin exhibited cytotoxicity at concentrations above 10 ^‒4^ M to 10 ^‒3^ M, which meant that both types of microglial cells had markedly decreased viability ( Figure 1A,B). Therefore, three different concentrations of calycosin (1×10 ^‒6^, 2×10 ^‒6^, and 4×10 ^‒6^ M) were used in the subsequent experiments. Our results revealed that OGD significantly reduced the viability of both BV2 and HAPI cells, while 1×10 ^‒6^ M to 4×10 ^‒6^ M calycosin increased the viability of OGD microglial cells in a dose-dependent manner ( Figure 1C,D).

### Effect of calycosin on the inflammatory response of microglia after OGD/R

**Figure 2:**
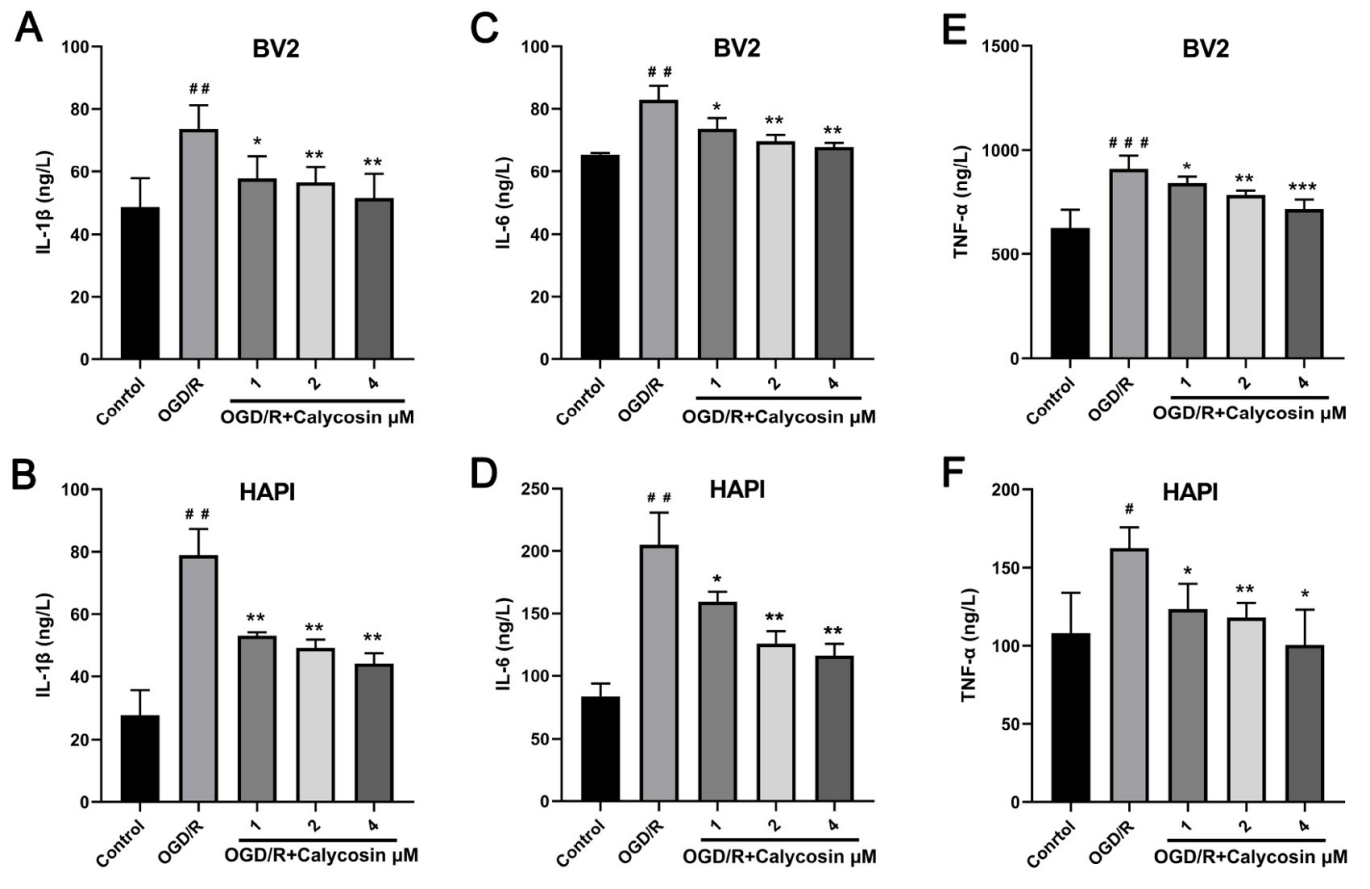
Figure 2 Effects of different concentrations of calycosin on inflammatory factors after OGD/R The expressions of inflammatory factors (IL-1β, IL-6, and TNF-α) in the BV2 supernatant (A–C) and the HAPI supernatant (D–F) were detected by ELISA. Data are expressed as the mean±SD, n ≥ 3. #P <0.05, ##P<0.01, ###P<0.001, the OGD/R group vs the control group. *P<0.05, **P<0.01, ***P<0.001, the calycosin treatment groups vs the OGD/R group.

To explore whether the effect of calycosin on microglia is related to the regulation of the inflammatory response, ELISA was used to detect the release of inflammatory factors. As shown Figure 2A, the IL-1β level in the supernatant of BV2 cells was approximately 48 ng/L in the control group, whereas it was significantly higher in the OGD/R group ( *P*<0.01), with an average concentration of 74 ng/L. Pretreatment with calycosin (1, 2, and 4 μM) effectively reduced the OGD/R-induced increase in IL-1β level ( *P*<0.05 or *P*<0.01), although the values were still higher than those in the control group. In addition, IL-6 and TNF-α produced by BV2 cells were also significantly increased in the OGD/R group compared with the control group. Pretreatment with calycosin decreased the secretion of the inflammatory factors IL-6 and TNF-α in BV2 cells after OGD/R in a dose-dependent manner ( Figure 2B,C). Similar trends were observed in HAPI cells ( Figure 2D‒F). These findings indicated that calycosin protected microglia after OGD/R by inhibiting the inflammatory response.

**Figure 3:**
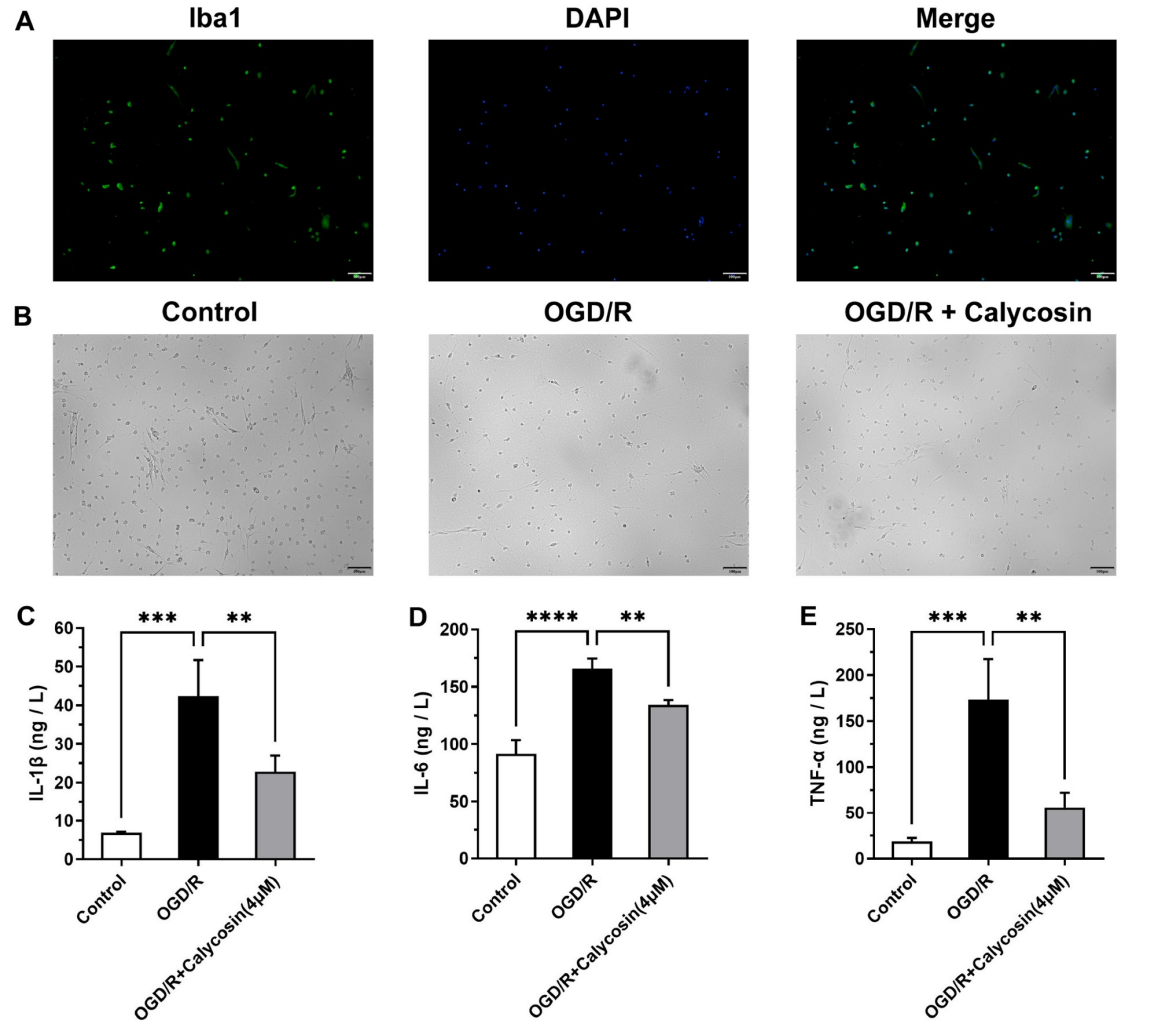
Figure 3 The identification and inflammatory factor detection of primary rat microglial cells (A) The purity of the primary rat microglial cells in culture was confirmed by immunofluorescence staining with the microglia marker Iba1 and DAPI (Scale bar: 100 μm). (B) OGD/R modelling and grouping of primary rat microglial cells under a microscope (scale bar: 100 μm). (C‒E) The expressions of inflammatory factors (IL-1β, IL-6 and TNF-α) in the primary rat microglial cell supernatant were detected by ELISA. Data are expressed as the mean±SD, n=3. *P<0.05, **P<0.01, ***P <0.001, ****P<0.0001.

Additionally, we further investigated whether calycosin can also reduce the inflammatory response in primary rat microglia after OGD/R. The immunofluorescence staining results showed that the purity of the primary rat microglial cells was more than 95%, which could be used for subsequent experiments ( Figure 3A). Under the inverted fluorescent microscope, normal cultured primary microglia displayed a slender, ramified morphology while at rest. After OGD/R, a decrease in the number of primary microglia, an increase in cell size, a reduction or disappearance of cell synapses, and a transition to an amebic morphology were observed, indicating a polarized state of the microglia. Notably, pretreatment with calycosin (4 μM) effectively increased the number of primary microglial cells and restored the ramified morphology in the OGD/R+calycosin group, suggesting that calycosin may improve the polarization state of microglia ( Figure 3B). The ELISA results indicated a significant increase in the release of inflammatory factors (IL-1β, IL-6, and TNF-α) by primary microglial cells in the OGD/R group compared to the control group. However, pretreatment with calycosin (4 μM) resulted in a noteworthy reduction in the secretion of these inflammatory factors in primary microglia following OGD/R ( Figure 3C‒E). These results suggested that calycosin protected microglia after OGD/R by inhibiting the inflammatory response.

### Effects of calycosin on the HMGB1/TLR4/NF-κB signaling pathway after OGD/R

**Figure 4:**
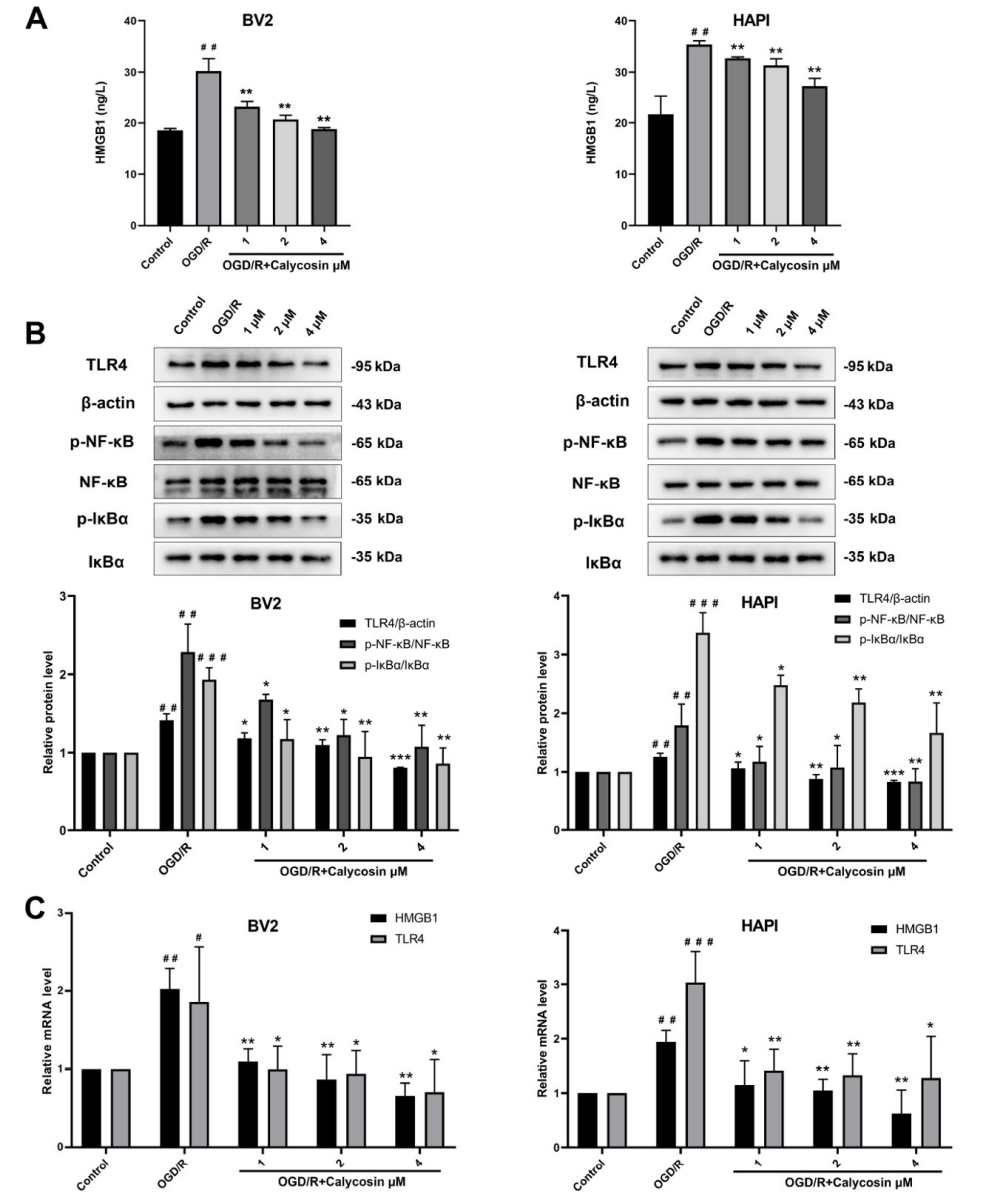
Figure 4 Calycosin inhibited the HMGB1/TLR4/NF-κB signaling pathway in microglia after OGD/R (A) Detection of HMGB1 content in supernatants by ELISA. (B) Protein expression levels of TLR4, p-NF-κB and p-IκBα. (C) HMGB1 and TLR4 mRNA expression levels. In western blot analysis, β-actin was used as the internal control for TLR4, NF-κB was used as the internal control for p-NF-κB, and IκBα was used as the internal control for p-IκBα. In qRT-PCR, β-actin was used as the internal control. Data are expressed as the mean±SD, n ≥ 3. #P <0.05, ##P<0.01, ###P<0.001, the OGD/R group vs the control group. *P<0.05, **P<0.01, ***P<0.001, the calycosin treatment groups vs the OGD/R group.

To explore whether the effect of calycosin on the cell inflammatory response is related to the HMGB1/TLR4/NF-κB signaling pathway, ELISA, western blot analysis, and qRT-PCR were used to detect the expressions of the pathway-related factors HMGB1, TLR4, NF-κB, and IκBα in both BV2 and HAPI cells. The ELISA results showed that in both the BV2 cells and the HAPI cells, the level of HMGB1 in the supernatant of the OGD/R group was higher than that of the control group ( *P*<0.01), and the level of HMGB1 in the supernatant of cells pretreated with different concentrations of calycosin (1, 2, and 4 μM) was lower dose-dependently than that of the OGD/R group ( *P* <0.01; Figure 4A). A comparison of TLR4 protein expression revealed that in BV2 cells and HAPI cells, the TLR4 protein expression level in the OGD/R group was higher than that in the control group ( *P*<0.01). Moreover, the TLR4 protein expression level in the OGD/R group was decreased after pretreatment with calycosin, particularly at a concentration of 4 μM ( *P*<0.001). The phosphorylation level of NF-κB in the OGD/R group was increased in both types of cells compared with that in the control group ( *P*<0.01), and the phosphorylation level of NF-κB was decreased after pretreatment with calycosin. The p-IκBα level in the OGD/R group was significantly higher than that in the control group ( *P*<0.001), and the phosphorylation level of IκBα was decreased after pretreatment with calycosin ( Figure 4B). In BV2 cells, the mRNA expression levels of HMGB1 and TLR4 in the OGD/R group were higher than those in the control group ( *P*<0.05). When the cells were pretreated with 1, 2, and 4 μM calycosin, the mRNA expression level was significantly lower than that in the OGD/R group. Similarly, in HAPI cells, the mRNA expression levels of HMGB1 and TLR4 in the OGD/R group were higher than those in the control group. Compared with those in the OGD/R group, the TLR4 and HMGB1 mRNA expression levels were decreased in the group pretreated with calycosin, particularly at concentrations of 2 and 4 μM ( Figure 4C). Our results suggested that calycosin might inhibit OGD/R-induced HMGB1/TLR4/NF-κB signaling pathway activation in microglia.

### Calycosin inhibits the inflammatory response of microglia via the HMGB1/TLR4/NF-κB signaling pathway

**Figure 5:**
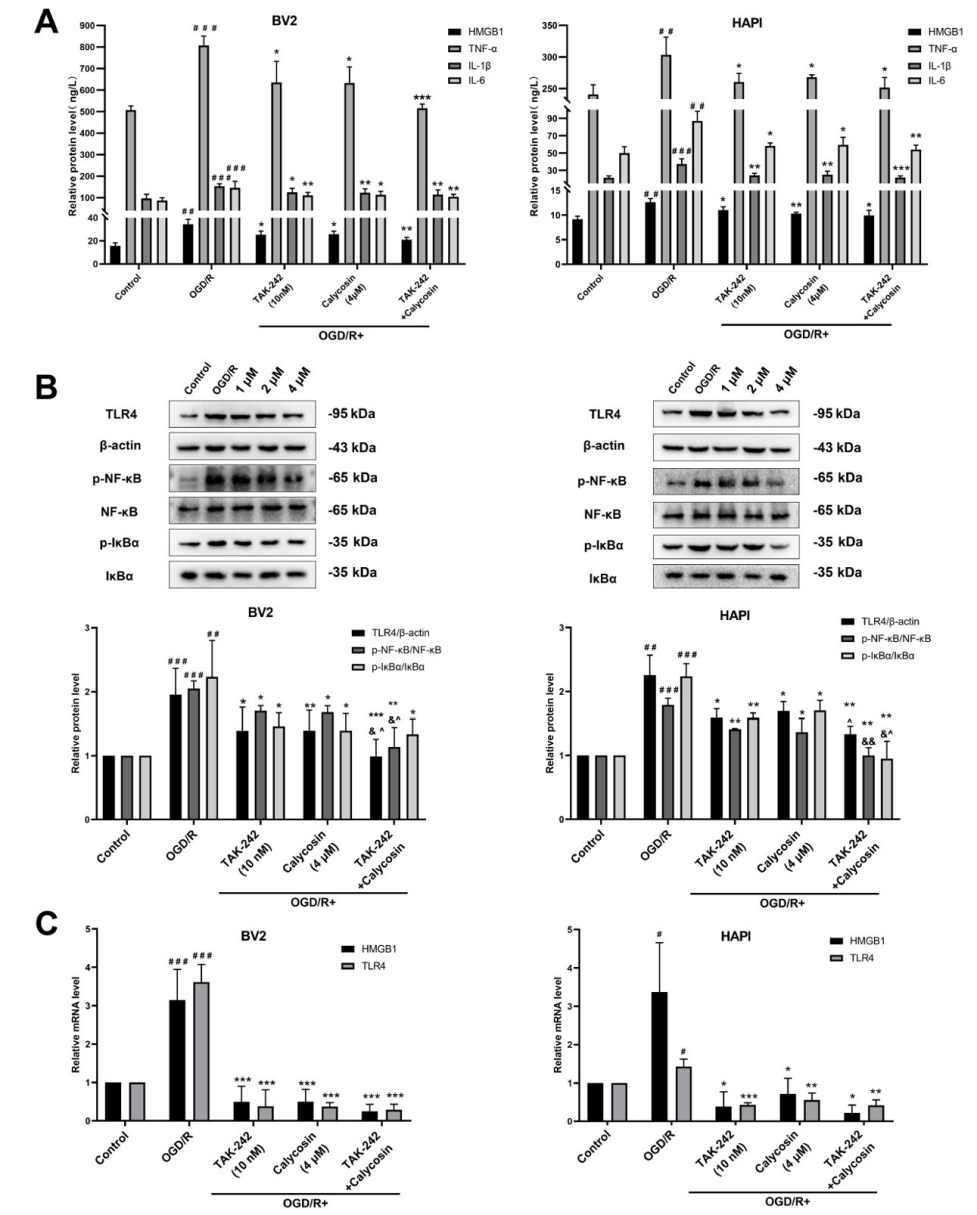
Figure 5 Neuroprotective effects of calycosin, TAK-242, and combined treatment on microglia after OGD/R (A) HMGB1, TNF-α, IL-1β and IL-6 levels in supernatants were detected by ELISA. (B) Protein expressions of TLR4, p-NF-κB, and p-IκBα. (C) HMGB1 and TLR4 mRNA expression levels. Data are expressed as the mean±SD, n ≥ 3. #P <0.05, ##P<0.01, ###P<0.001, the OGD/R group vs the control group. *P<0.05, **P <0.01, ***P<0.001, the calycosin and/or TAK-242 treatment groups vs the OGD/R group. &P<0.05, &&P<0.01, the TAK-242+calycosin group vs the TAK-242 group. ^P<0.05, ^^P<0.01, the TAK-242+calycosin group vs the calycosin group.

To further confirm the anti-inflammatory mechanism of calycosin on microglia after OGD/R, the HMGB1/TLR4/NF-κB signaling pathway was inhibited by TAK-242 (a specific inhibitor of TLR4). According to the manufacturer’s recommendations and our preliminary results, we chose a concentration of 10 nM TAK-242 and 4 μM calycosin for the subsequent experiments. The ELISA results revealed that when TLR4 was inhibited, the HMGB1 and inflammatory factors (TNF-α, IL-1β, and IL-6) produced by OGD microglia were significantly reduced. As illustrated in Figure 5A, calycosin also reduced the production of HMGB1 and inflammatory factors in both BV2 and HAPI cells, and the combined effect of TAK-242 and calycosin was more obvious. The western blot analysis results demonstrated that the protein expression levels of TLR4 and the phosphorylation of NF-κB and IκBα significantly were decreased when TLR4 was specifically inhibited by TAK-242. Similarly, the protein expression of TLR4 and the phosphorylation levels of NF-κB and IκBα were markedly reduced by calycosin in both microglia cells after OGD/R. These effects were further enhanced in the combination administration group ( Figure 5B). The qRT-PCR results showed that the mRNA levels of HMGB1 and TLR4 were significantly increased in the OGD/R group compared with those in the control group. Both TAK-242 and calycosin significantly reduced the mRNA levels of HMGB1 and TLR4 in these two microglia cell lines after OGD/R, and the same effects were observed in the combined administration group ( Figure 5C). These results suggested that calycosin might attenuate the inflammatory response of OGD microglial cells by inhibiting the HMGB1/TLR4/NF-κB signaling pathway.

### Molecular docking analysis of the TLR4 receptor and its candidate ligands TAK-242 and calycosin

**Figure 6:**
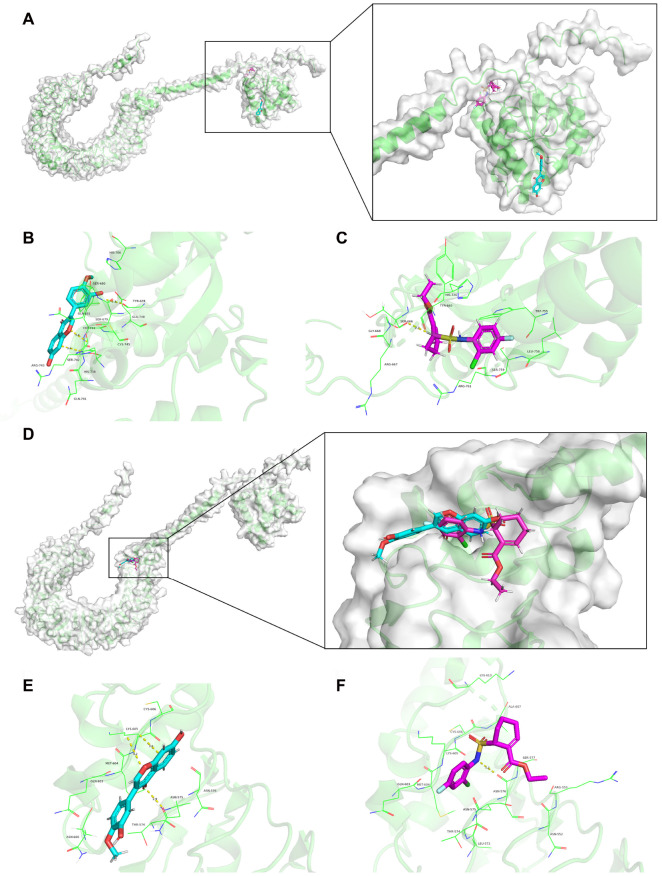
Figure 6 Molecular docking model of the TLR4 receptor and its ligand (calycosin/TAK-242) (A) Global and local views of mouse TLR4 receptor docking with its ligand (calycosin/TAK-242). (B) The binding sites and interactions between calycosin and mouse TLR4 receptor. (C) The binding sites and interactions between TAK-242 and mouse TLR4 receptor. (D) Global and local views of rat TLR4 receptor docking with its ligand (calycosin/TAK-242). (E) The binding sites and interactions between calycosin and rat TLR4 receptor. (F) The binding sites and interactions between TAK-242 and rat TLR4 receptor.

To determine whether calycosin and TAK-242 exhibit inhibitory effects by binding to distinct sites on TLR4, a molecular docking analysis was conducted on the TLR4 receptor and its candidate ligands. The results indicated that each candidate binds to its protein target through visible hydrogen bonds and strong electrostatic interactions. Moreover, the hydrophobic pockets of each protein target are occupied successfully by the two candidate drugs. For mouse TLR4 (Q9QUK6), two candidates (TAK-242 and calycosin) have low binding energies of ‒6.4 and ‒6.8 kcal/mol. For rat TLR4 (Q9QX05), both of them also have low binding energies of ‒6.5 and ‒6.3 kcal/mol. These results indicate that both TAK-242 and calycosin are highly stable in binding to TLR4. To validate the interaction results, TAK-242 and calycosin were docked into the active site cavity of TLR4. In the case of mouse TLR4, TAK-242 poses in the active site by interacting with the amino acid residues at Ser666, His726, Tyr665, Arg761, Ser759, Gly668, Leu758, Trp755, and Arg667. Calycosin shares Tyr678, Ser742, Trp744, Ser680, Gln741, His706, Gln681, Cys745, Ser679, Glu748, His738, and Arg743 as interacting residues ( Figure 6A‒C). The docking simulation results suggest that tak-242 and calycosin have different binding regions of mouse TLR4. For rat TLR4, TAK-242 interactes with Asn576, Ser577, Asn575, Lys605, Ala607, Gln603, Lys613, Thr574, Asn552, Arg553, Leu573, Met604, and Cys606. Meanwhile, calycosin also shows high affinity via interactions with Asn575, Lys605, Asn600, Gln603, Thr574, Asn576, Met604, and Cys606, indicating that the binding sites of rat TLR4 for TAK-242 do not exactly match those for calycosin ( Figure 6D‒F). Collectively, the combined treatments of calycosin and TAK-242 likely enhance the inhibitory effects by acting on different active sites of TLR4.

## Discussion

Many studies have revealed that the inflammatory response plays a key role in the pathophysiology of cerebral ischaemia‒reperfusion injury, and the activation of the HMGB1/TLR4/NF-κB signaling pathway has been found to be involved in cerebral ischemia-induced inflammation [ 14– 17]. In the current study, we demonstrated that the phytoestrogen calycosin inhibited the HMGB1/TLR4/NF-κB signaling pathway and reduced proinflammatory factors in microglia after OGD/R, suggesting that the inflammatory response induced by CIRI might be prevented by calycosin.

As an endogenous damage-associated molecular pattern (DAMP), HMGB1 contributes to inflammation and the innate immune response in various neurological diseases [ 18, 19]. In the inflammatory state, HMGB1 can be passively released from necrotic cells or actively secreted by immunocompetent cells, such as monocytes, macrophages, and microglia [2]. TLR4 is a pattern recognition receptor (PRR) that recognizes its endogenous ligand HMGB1 to promote neuroinflammation. When CIRI occurs, HMGB1 binding to TLR4 activates myeloid differentiation factor (MyD88), resulting in the phosphorylation of NF-κB (IκB). Immediately, NF-κB dissociates from the complex and translocates into the nucleus. In the nucleus, NF-κB binds with specific DNA sequences and initiates gene transcription and the expression of inflammatory reaction proteins, thus promoting ischaemia‒reperfusion injury [20]. Several recent studies have reported that activating the HMGB1/TLR4/NF-κB signaling pathway is responsible for the exacerbation of CIRI, whereas inhibiting this signaling pathway alleviates injury [ 21, 22]. Our data confirmed that TLR4 protein expression was increased, the phosphorylation of NF-κB and IκBα was enhanced, and the production of HMGB1 and inflammatory factors was increased in OGD/R microglia, showing the effect of HMGB1/TLR4/NF-κB signaling pathway activation. However, this effect could be reversed by the TLR4 inhibitor TAK-242.

Microglia, as resident immune cells in the central nervous system, are usually in a quiescent state. When stimulated, microglia can be polarized into M1 (proinflammatory phenotype) or M2 (anti-inflammatory phenotype). M1 microglia mainly secrete proinflammatory factors, such as IL-1β, iNOS, and TNF-α, which induce oxidative damage and aggravate the inflammatory response. M2 microglia mainly secrete anti-inflammatory factors, such as IL-10 and transforming growth factor-β (TGF-β), which exert their anti-inflammatory, repair, and regeneration effects [23]. Recent *in vitro* and *in vivo* studies revealed that bioactive natural products or drug molecules that promote microglial polarization from the M1 phenotype toward the M2 phenotype can significantly alleviate cerebral ischaemia‒reperfusion injury [ 24, 25]. Our current study indicated that OGD/R activated microglia to transform from quiescent microglia to the proinflammatory M1 phenotype, thereby releasing a large amount of HMGB1 and proinflammatory cytokines (TNF-α, IL-1β, and IL-6).

Recently, calycosin has attracted increasing attention for its potential neuroprotective role in CIRI [ 26, 27]. Many mechanisms have been proposed to account for the neuroprotection of calycosin. Guo *et al*. [28] reported that calycosin significantly reduces the neurologic deficit score and the infarct volume in a rat MCAO model and that the neuroprotective mechanism is related to its antioxidant effect. Another recent study demonstrated that calycosin treatment ameliorated neurological injury by modulating the brain-derived neurotrophic factor (BDNF)-tyrosine kinase receptor B (TrkB) signaling pathways and microglial activation in poststroke rats [29]. Our previous studies showed that calycosin upregulates the expressions of ER-α, miR-375, P62, NBR1 and bcl-2 and downregulates the expression of RASD1. The neuroprotective effect of calycosin on MCAO model rats is achieved through various mechanisms, including anti-inflammatory, anti-apoptosis, and anti-autophagy mechanisms [ 11 , 30]. In this study, we found that pretreatment with calycosin could significantly reduce the release of HMGB1 and the expression of TLR4, inhibit the phosphorylation level of NF-κB and IκBα in OGD/R microglia, and weaken the tendency of microglia to proinflammatory activation. Furthermore, we observed that when TAK-242 was used to inhibit TLR4, calycosin still markedly inhibited the activation of the HMGB1/TLR4/NF-κB signaling pathway, suggesting that calycosin, as a pleiotropic neuroprotective agent, might inhibit the inflammatory response through multiple mechanisms.

In summary, our study demonstrated that calycosin suppressed the activation of the HMGB1/TLR4/NF-κB signaling pathway and proinflammatory depolarization in OGD/R microglia, contributing to its neuroprotective effects. The findings of this study indicated that calycosin might be a therapeutic candidate for ischemic stroke because of its anti-inflammatory properties. However, the clinical application of calycosin has yet to be studied in depth.
